# Clinical diagnosis and treatment recommendations for immune checkpoint inhibitor‐related adverse reactions in the nervous system

**DOI:** 10.1111/1759-7714.13266

**Published:** 2019-12-10

**Authors:** Jiayu Shi, Jingwen Niu, Dongchao Shen, Mingsheng Liu, Ying Tan, Yi Li, Yangyu huang, Liying Cui, Yuzhou Guan, Li Zhang

**Affiliations:** ^1^ Neurology department, Peking Union Medical College Hospital Peking Union Medical College and Chinese Academy of Medical Sciences Beijing China

**Keywords:** Adverse reaction of nervous system, glucocorticoid, immune checkpoint inhibitor

## Abstract

Immune checkpoint inhibitors (ICIs) can cause adverse reactions in the nervous system. The incidence rate is 0.1%–12% and 80% of nervous system adverse reactions occur within the first four months of application. ICIs can cause diseases of various parts of the nervous system including central nervous system diseases such as aseptic meningitis, meningeal encephalitis, necrotizing encephalitis, brainstem encephalitis, transverse myelitis, etc., and peripheral neuropathy such as cranial nerve peripheral neuropathy, multifocal nerve root neuropathy, Guillain‐Barré syndrome, spinal nerve root neuropathy, myasthenia gravis, myopathy, etc. For these complications of the nervous system, diagnosis could be difficult. Physicians require a specific collection of nervous system symptoms and signs, combined with supplementary examinations including imaging, cerebrospinal fluid cytology, EEG or electromyography in order to exclude infection or malignant tumor before reaching a final diagnosis. With regard to treatment, ICIs should be discontinued in severe cases, and large doses of glucocorticoid or gamma globulin administered, and supportive treatment may be necessary. If severe adverse reactions of the nervous system occur, the prognosis could be poor.

## Introduction

Immune checkpoint inhibitors (ICIs) are aimed at receptor‐1 (PD‐1) and cytotoxic T‐ lymphocyte‐associated antigen‐4 (CTLA‐4) antibodies. There are two key pathways in the activation process of immune T‐cells: CTLA‐4/B7‐1/2 and PD‐1/PD‐L1, which makes tumor associated antigen unable to trigger the activation signal pathway and has an inhibitory effect on a variety of tumors. So far, the US Food and Drug Administration (FDA) has approved six kinds of ICIs which include O‐drug Opdivo (nivolumab, avelumab, Opdivo); K‐drug Keytruda (pembrolizumab, pabolizumab, coreda); T‐drug Tecentriq (atezolizumab, atzumab, tersanqi); I‐drug Imfinzi (duravalumab, duvalimab); B‐drug Bavencio (avelumab); L‐drug Libtayo (cemiplimab‐rwlc [EpimAb Biotherapeutics (HK) Limited]). ICIs are now used to treat various types of cancers including bladder cancer, head and neck cancer and different types of advanced NSCLC. With the expansion of use of these drugs, drug‐related adverse reactions have been reported. The specific immune‐related adverse reactions caused by ICIs are called immune‐related adverse effects (irAEs). In this report, the incidence rate, clinical manifestations, diagnosis and treatment of the nervous system irAEs are summarized, in order to improve the knowledge, early warning and treatment measures for neurologists and oncologists.

## Incidence of adverse reactions of the nervous system

There are relatively few reports on the incidence of adverse reactions in the nervous system to ICIs. A retrospective study which included 59 clinical studies and 920 cases reported that the incidence of irAEs in the nervous system caused by CTLA‐4 inhibitors was 3.8%, PD‐1 inhibitors 6.1%, and CTLA‐4 inhibitors combined with PD‐1 inhibitors was 12.0%.^1^ A phase III clinical trial (eortc18071) reported that the incidence of nervous system irAEs in adult anti‐CTLA‐4 group was 4%.^2^ Severe (grade 3–4) neurological irAEs occurred in 1.9% of patients with anti‐CTLA‐4, 0.2%–0.4% of patients with anti‐PD‐1 and 0.1%–1% of patients with anti‐PD‐L1.^3^ Cuzzubbo *et al*.^1^ analyzed 27 patients who received ICI treatment and developed nervous system irAEs. The results showed that the median time of occurrence of nervous system irAEs was six weeks (1–74 weeks). All cases were acute or subacute and related to tumor response. Spain *et al*.^4^ and Zimmer *et al*.^5^ reported that 80% and 75% of patients, respectively with irAEs after ICI treatment occurred in the first four months of immunotherapy. It can therefore be seen that irAEs of the nervous system mostly occur in the induction stage of patients' ICI treatment. Monitoring the occurrence of irAEs of the nervous system in the first four months is therefore important. Based on the above results, the nervous system irAEs are common adverse reactions (≥1%) to ICIs and should be given due attention. A summary of nervous system‐related irAEs reported in the literature are shown in Table 1.

**Table 1:** Summary of nervous system immune‐related adverse effects (irAEs)

ICIs	Central nervous system irAEs	Peripheral nervous system irAEs
Anti CTLA‐4 Ipilimumab	Transverse myelitis	Pericranial neuropathy
	Aseptic meningitis	Polyradiculoneuropathy
	Meningoencephalitis	Guillain‐Barré syndrome
	Necrotizing encephalitis	Spinal radiculopathy
	Brainstem encephalitis	myasthenia gravis
Anti PD‐1	Limbic encephalitisInflammatory demyelinating disease of central nervous systemBrainstem encephalitis	Guillain‐Barré syndromemyasthenia gravis

## Adverse central nervous system reactions to ICIs

Patients who are treated with ICIs may have nonspecific neurological symptoms including dizziness, headache, drowsiness, weakness, mental malaise, dullness and other reactions. For this kind of phenomenon, rest and symptomatic treatment should be given to maintain the balance of water and electrolytes.

### Pituitary inflammation

The clinical manifestations of pituitary inflammation can cause neurological symptoms, especially headache, fatigue and weakness. Up to 10% of patients treated with Ipilimumab developed hypophysitis. Anti PD‐1/PD‐L1 is rarely involved. Generally, pituitary inflammation occurs 2–3 months after initiation of treatment with ICIs.^6^ Diagnostic tests include serological tests related to the endocrine axis (corticotropin, cortisol, thyrotropin, F‐T4, luteinizing hormone, follicle stimulating hormone, testosterone/estrogen and electrolytes). Cranial magnetic resonance imaging (MRI) may show enlargement of the pituitary gland and other possible differential diagnosis, such as tumor metastasis, should be excluded when considering the diagnosis. In terms of treatment, it is not recommended to use large doses of steroids (except for patients with obvious symptoms), because such treatment will not improve the prognosis. In most cases, the destruction of the pituitary gland is irreversible and requires long‐term hormone replacement therapy. Treatment with ICIs can be resumed after hormone replacement therapy.

### Immune‐mediated encephalitis

Immune‐mediated encephalitis caused by ICIs has various and atypical symptoms and is difficult to diagnose. According to a recent study by Larkin *et al*. immune‐mediated encephalitis usually occurs 55 days (18–297 days) after treatment with ICIs. Approximately 0.2% of the patients receiving PD‐1 treatment developed immune‐mediated encephalitis, including marginal lobe encephalitis, brainstem encephalitis and necrotizing encephalitis.^7^, ^8^, ^9^ The clinical manifestations of immune‐mediated encephalitis are nonspecific but the main symptoms are headache, fever, mental disorder, memory disorder, drowsiness, hallucination, seizure, neck stiffness, mental state decline, attention impairment and disorientation.^10^, ^11^, ^12^ Once these related symptoms are apparent it is necessary to evaluate the patient with a cranial MRI and CSF examination. An MRI will highlight limited diffusion of the limbic system^9^ or large lesions with mild enhancement.^8^, ^10^ In addition, the level of IgG in CSF is increased which indicates the effect of ICIs on the function and differentiation of B cells.^13^ Pathological examination does not usually reveal any definitive results. According to previous reports, the encephalitis caused by irAEs may be shown as extensive demyelination, edema and necrosis, or evidence of lymphocytic infiltration around blood vessels and brain parenchyma.^6^, ^7^, ^8^

### Aseptic meningitis

Aseptic meningitis is a rare side effect which has been previously reported when patients were treated with Ipilimumab. It usually occurs between the first and seventh week after the administration of ICIs. The main symptoms include stiff neck, fever and headache. Cerebrospinal fluid was sterile fluid, while mainly lymphocytic infiltration was reported. MRI showed meningeal enhancement. Steroid therapy is usually effective.^14^

### Others

Inflammatory demyelinating diseases such as multiple sclerosis, including optic neuritis, transverse myelitis and acute tumor demyelinating disease, have been reported during treatment with Ipilimumab. Abdallah *et al*. reported a case of transverse myelitis after the application of Ipilimumab. The main clinical manifestations were paraplegia, urinary retention and lower limb sensory disturbance. Whole spinal cord enhanced MRI showed that T2 phase high enhancement was apparent in the cervical spinal cord, thoracic spinal cord, conus, cauda equina and sacral nerve root. Following lumbar puncture the cerebrospinal fluid (CSF) showed an increase in leukocyte count, protein 310 mg/dL and glucose 27 mg/dL. Pathological examination of the lumbar spinal cord showed necrosis accompanied by infiltration and aggregation of tissue cells and lymphocytes around the blood vessels, no damage to the blood vessel wall, and no thrombosis formation was detected.^15^

## Adverse reactions of peripheral nervous system to ICIs

### Peripheral neuropathy

ICI‐induced peripheral neuropathy occurs in less than 1% of patients, which is a rare complication. Supakornnumporn and Katirji^16^ described five patients with Guillain‐Barré syndrome (GBS) after treatment with ICIs. A total of 80% of patients showed corresponding clinical manifestations in the third treatment cycle, which mainly included sensory loss, paraplegia, weakness, sensory abnormality, numbness, dysphagia, etc. The cerebrospinal fluid of the patient indicated the separation of protein and cells, and the electromyogram indicated multiple peripheral nerve demyelination. The treatment included immunoglobulin therapy, glucocorticoid treatment, immunoglobulin and glucocorticoid combined, tacrolimus application, plasma exchange treatment, etc. In terms of prognosis, 40% of the patients died after treatment, 40% of the patients improved significantly, and 20% of the patients maintained their symptoms.

In addition, three cases^17^, ^18^, ^19^ reported facial paralysis after ICI treatment. The clinical manifestations of the patients were mainly typical peripheral facial paralysis which could be accompanied by diffuse papules, but there was no obvious abnormalities in MRI and CSF. In terms of treatment, glucocorticoid treatment was reported to be effective.

### Myasthenia gravis

It is generally thought that treatment with ICIs can lead to potential autoimmune disorders. At present, it is believed that Ipilimumab can induce T‐cells to produce acetylcholine receptor antibody, leading to the occurrence or deterioration of myasthenia gravis. Makarious *et al*.^20^ reported 23 cases of ICI‐related myasthenia gravis, 70% of which were new cases (13 received anti‐PD‐1, 4 anti‐CTLA‐4 and 3 combined). The incidence of myasthenia gravis was 0.12% in a large group of patients treated with Ipilimumab. Clinical manifestations include ptosis, diplopia, muscle weakness, dyspnea and dysphagia. Acetylcholine receptor antibody was positive in 59% of patients. Myositis occurred in nine cases. Most patients began to show symptoms 7–11 weeks after treatment. Approximately one third of the patients died after active treatment including steroid, intravenous immunoglobulin (IVIG) or plasma exchange.

### Inflammatory myopathy

Myopathy seems to be the most common neurological irAEs against PD‐1/PD‐L1, while the incidence of anti‐CTLA‐4 is relatively low. The most common types are necrotic autoimmune myositis, dermatomyositis and polymyositis.^4^, ^21^, ^22^, ^23^ In addition, rare cases of myopathy such as orbital myositis and eosinophilic fasciitis have been reported.^24^, ^25^ Common symptoms include muscle pain, weakness of the proximal extremities, difficulty speaking/swallowing, ptosis or weakness of the oculomotor muscles. Moreira *et al*. described 19 cases of ICI‐induced myositis, 32% of which were associated with myocarditis and 5% with myasthenia gravis, all of which received at least anti‐PD‐1 treatment. Some patients had respiratory distress which was related to diaphragm involvement. Laboratory tests often showed elevated creatine kinase.^24^ Electrophysiological examination showed myogenic damage. Necrotic muscle fibers and inflammatory changes were seen on muscle biopsy.^24^ Large doses of corticosteroids and discontinuation of ICIs usually improved the symptoms and most of the patients recovered completely.

## Diagnosis and corresponding treatment of nervous system irAEs of ICIs

**Figure 1:**
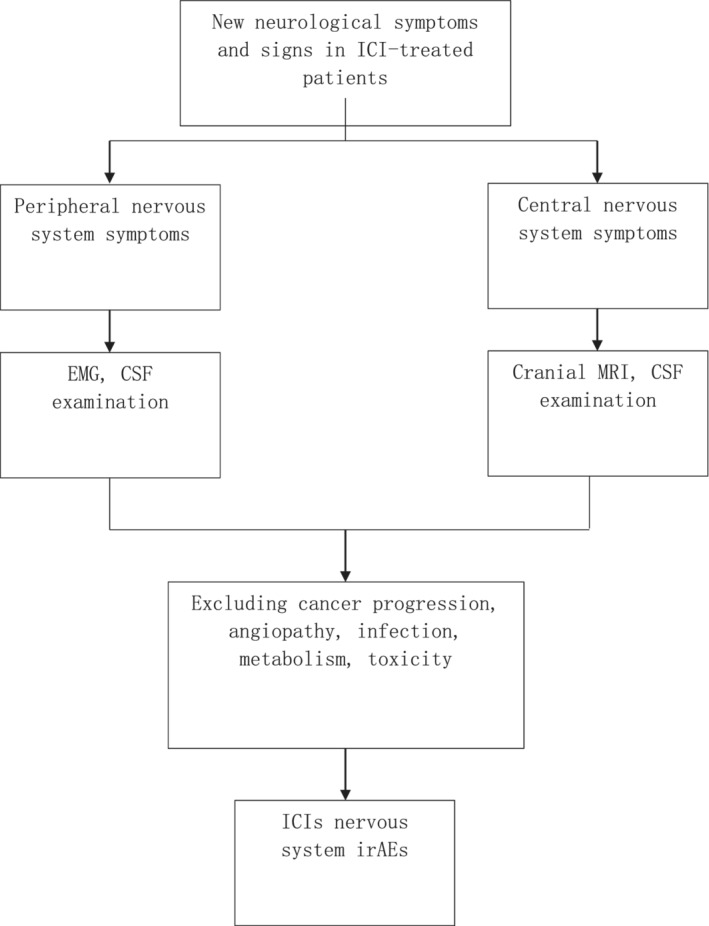
Diagnostic flow‐chart of immune checkpoint inhibitors (ICIs) nervous system immune‐related adverse effects (irAEs).

The diagnostic process of neurological irAEs of ICIs is shown in Figure 1. According to the clinical manifestations, patients with new moderate to severe neurological signs or symptoms after treatment with ICIs can immediately be initiated into the diagnosis process. Cranial MRI, cerebrospinal fluid examination, and electromyographic examination can be carried out in order to exclude other causes such as vascular disease, progressive tumor disease (brain metastasis, leptinoma or spinal cord compression), infection, paraneoplastic syndrome or toxic/metabolic factors. Neurological irAEs of ICIs can be diagnosed only after all possible interferences have been excluded.^4^ Rapid diagnosis and treatment is essential because neurological irAEs can lead to serious sequelae or death. A multi‐disciplinary team should participate in decision management for patients treated with ICIs because on the one hand, stopping tumor treatment may reduce the efficiency of drug treatment; on the other hand, severe nervous system irAEs will shorten the survival period of patients. If necessary, lumbar puncture and MRI should be conducted to further clarify the cause of the disease and carry out further immunotherapy as early as possible.^3^ The differential diagnosis of central nervous system and peripheral neurological irAEs of ICIs and the corresponding auxiliary examination and clinical treatment are as follows.

### Diagnosis and treatment process of ICIs' irAEs in the central nervous system

#### Encephalitis

With regard to a differential diagnosis, encephalitis must be differentiated from infectious diseases, metabolic diseases, brain metastasis, cerebrovascular diseases such as cerebral hemorrhage, cerebral infarction and metastatic tumor of the pia mater. With regard to the auxiliary examination, serological examination including electrolytes, blood sugar, total protein, serum protein electrophoresis and virological serum examination, head and spinal MRI scans, cerebrospinal fluid examination, WBC count, protein, sugar, chloride level, HSV and other virus qualitative and CSF cytology should be carried out. Disease treatment needs to be evaluated according to the severity level, which can be graded as G1–G4 level. G1: mild symptoms without limiting daily life; G2: new moderate or severe neurological signs or symptoms; G3–G4: encephalitis with mental and behavioral abnormalities. For grade G1, it is recommended to maintain ICI treatment and start the diagnostic process. If there is no improvement or deterioration of symptoms, the ICI should be stopped permanently. For grade G2, ICI treatment can be maintained temporarily, and the symptoms and signs should be monitored closely, except for viral or bacterial infection (except for empirically using antiviral drugs or antibiotics before infection). For grade G3–G4 patients, the ICI should be stopped permanently, except for a large dose of glucocorticoid (0.5–1 mg/kg/day) after infection.

#### Meningitis

In terms of a differential diagnosis, meningitis must be differentiated from infection, metabolism, tumor meningeal dissemination and tumor metastasis. In terms of examination, it is suggested that an MRI of the head and spinal cord be performed and cerebrospinal fluid examination following lumbar puncture to clarify WBC count, protein, sugar, chloride level, HSV and other virus qualitative cytology be completed. In terms of treatment, glucocorticoids can be used in large doses after a bacterial or viral infection has been excluded.

#### Transverse myelitis

Transverse myelitis must be differentiated from spinal cord metastasis, spinal cord compression and infectious diseases. It is suggested that serum vitamin B12, TSH, HIV, syphilis, Ana, anti‐Ro and anti‐La antibodies, AQP4 antibody test, head and spinal MRI, WBC count evaluation, protein, sugar, chloride level, HSV and other virus qualitative cytology are carried out. In terms of treatment, a large dose of glucocorticoid can be given. If hormone treatment is ineffective, IVIG or plasma exchange should be considered.

### Diagnosis and treatment process of ICIs' irAEs of peripheral nervous system

#### Polyneuropathy

With regard to a differential diagnosis, polyneuropathy should be differentiated from metabolism and poisoning (chemotherapy and vitamin deficiency). If the cranial nerve is involved, meningeal metastasis should first be excluded. In terms of auxiliary examination, it is suggested that the serum electrolyte, vitamin B12, electromyography (EMG), protein evaluation and cells in the cerebrospinal fluid following lumbar puncture are examined, and a cranial MRI performed. The disease treatment needs to be evaluated according to the severity level, which is graded into G1–G4 levels. G1: refers to mild symptoms without limitation of daily life; G2: refers to mild symptoms, affecting daily life (any cranial nerve involvement is classified as G2); G3–G4: refers to severe limitation of daily life or respiratory involvement. For grade G1, it is suggested that ICI treatment is maintained at a low dose and the patient observed for a change in clinical symptoms; for grade G2, it is suggested that ICI treatment is suspended, and the change of symptoms monitored, or glucocorticoid 0.5–1 mg/kg given and symptomatic treatment; for grade G3–G4, it is suggested that the patient be hospitalized, ICI treatment suspended and a high dose of glucocorticoid 2 mg/kg given.

#### Guillain Barré syndrome

In terms of differential diagnosis, Guillan Barré syndrome should to be differentiated from spinal cord compression, infectious diseases (Lyme, HIV, HSV, HZV), metabolic diseases, side effects of drugs, chronic inflammatory demyelinating polyneuropathy (CIDP) and vasculitis. It is suggested that an EMG and lumbar puncture should be performed to determine whether there are protein‐cell‐separation symptoms. Clinicians should be aware of the potential for respiratory muscle involvement and blood gas analysis and a lung function test should be conducted. If diplopia or ocular dyskinesia occur, a ganglioside antibody (GQ1b) test will be necessary. In terms of treatment, high‐dose glucocorticoid is the first choice, and the symptoms at the initial stage of treatment should be monitored since symptom deterioration may occur in this stage; if there is no improvement, plasma exchange or intravenous immunoglobulins (IVIg) can be used. The degree of autonomic nervous dysfunction or respiratory dysfunction determines whether further treatment in ICU is required.

#### Myasthenia gravis

With regard to a differential diagnosis, myasthenia gravis should be differentiated from metabolic myositis, toxic‐induced myasthenia syndrome and polymyositis. It is suggested that an acetylcholine receptor antibody (AChR‐ab) or muscle‐specific tyrosine kinase (MuSK‐ab) test should be performed, and electrophysiology, repetitive nerve stimulation (RNS) as well as single fiber electromyography are required for diagnosis. In terms of treatment, it is suggested that ICI treatment should be discontinued and high‐dose glucocorticoid treatment given; if the initial treatment is invalid, IVIg or plasma exchange can be applied.

#### Myositis

Myositis must be differentiated from glucocorticoid myositis and drug‐related myositis. Serum CK test and EMG are required to achieve this together with a muscle biopsy, if necessary. In terms of treatment, high‐dose glucocorticoids should be administered. If respiratory muscle is involved, blood oxygen should be monitored closely. Monitoring the patient for possible myocardial involvement is also essential.

## Prognosis of ICIs' irAEs in the nervous system

In terms of prognosis, researchers comprehensively searched the clinical trials, case reports and other literature on the application of ICIs in Bristol Myers Squibb safety database from 4 January 2016 to 3 July 2016, and determined that there were 28 cases of encephalitis related to single or combined treatment with ICIs, among which the mortality of encephalitis in single and combined treatment groups were 1/19 (5%) and 1/9 (11%), respectively.^26^ In conclusion, the mortality rate of encephalitis in a combination treatment group was significantly higher than that in a single drug treatment group.

The application of ICIs in cancer treatment has brought new hope to oncologists and patients. Once neurological irAEs appear after treatment with ICIs, if the treatment is not appropriate, it may endanger the life of patients. Early recognition and early treatment are critical. Most of the neurological irAEs of ICIs are acute or subacute, which may occur at any time during, or after, treatment. Prevention or reduction of neurological irAEs remains a key challenge for clinicians. However, the current consensus guidelines are still based on empirical data. Prospective clinical trials will be the key to the future research of neurological irAEs.

## Disclosure

No authors report any conflict of interest.
